# Supplementary material for: Genetic Loss of miR-205 Causes Increased Mammary Gland Development
Source: Noncoding RNA. 2023 Dec 31;10(1):4. doi: 10.3390/ncrna10010004 (PMC10801544; doi:10.3390/ncrna10010004)
Supplement: Supplementary file 1 [file ncrna-10-00004-s001.zip › ncrna-2733483-supplementary.pptx]

## Slide 1
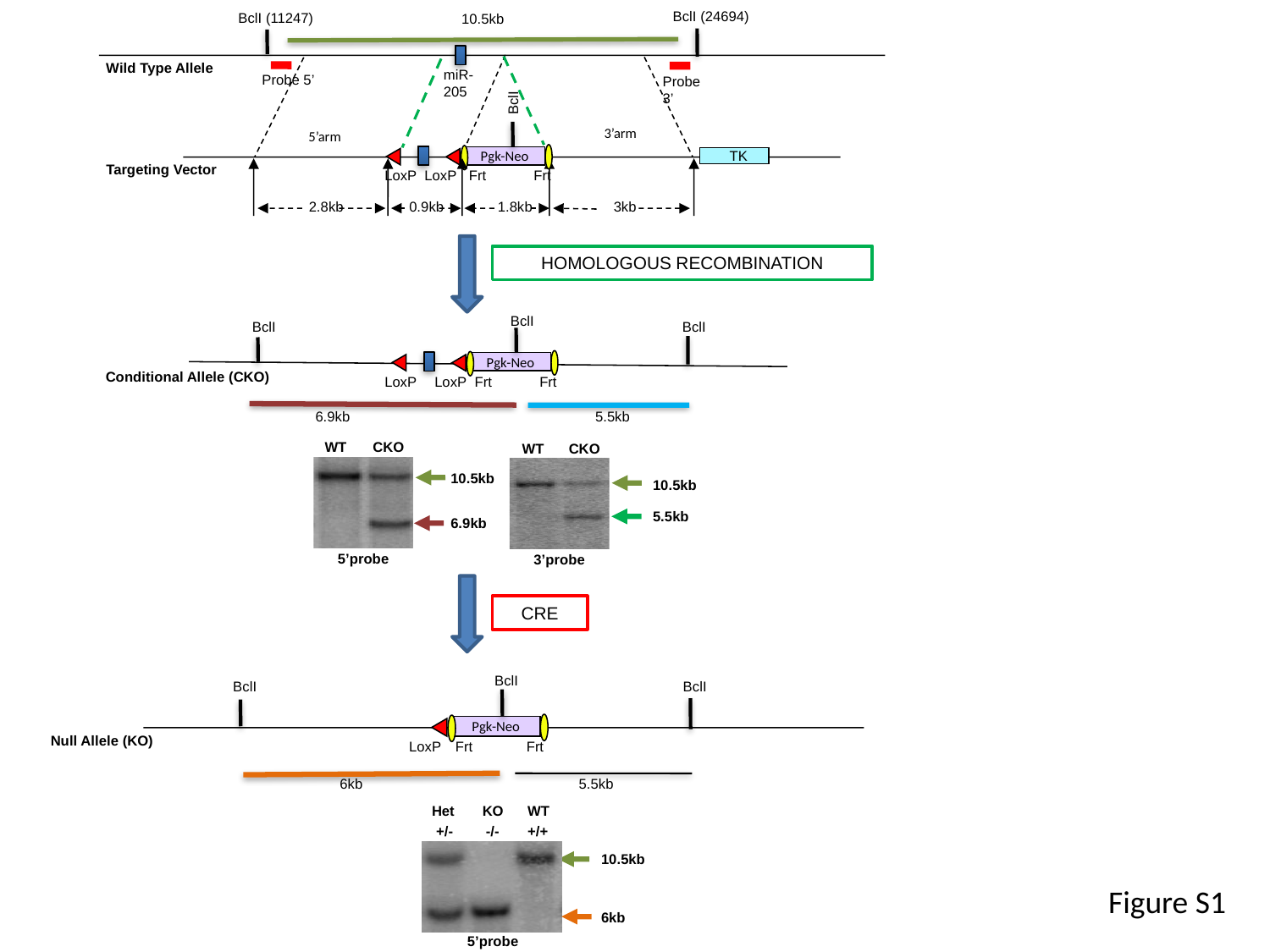

BclI (24694)
BclI (11247)
10.5kb
BclI
Wild Type Allele
miR-205
Probe 5’
Probe 3’
3’arm
5’arm
TK
Pgk-Neo
Targeting Vector
LoxP
LoxP
Frt
Frt
2.8kb
0.9kb
1.8kb
3kb
HOMOLOGOUS RECOMBINATION
BclI
BclI
BclI
Pgk-Neo
Conditional Allele (CKO)
LoxP
LoxP
Frt
Frt
6.9kb
5.5kb
WT
CKO
WT
CKO
10.5kb
6.9kb
5’probe
3’probe
10.5kb
5.5kb
CRE
BclI
BclI
BclI
Pgk-Neo
Null Allele (KO)
Frt
Frt
LoxP
5.5kb
6kb
+/-
-/-
+/+
5’probe
10.5kb
6kb
Het KO WT
Figure S1

## Slide 2
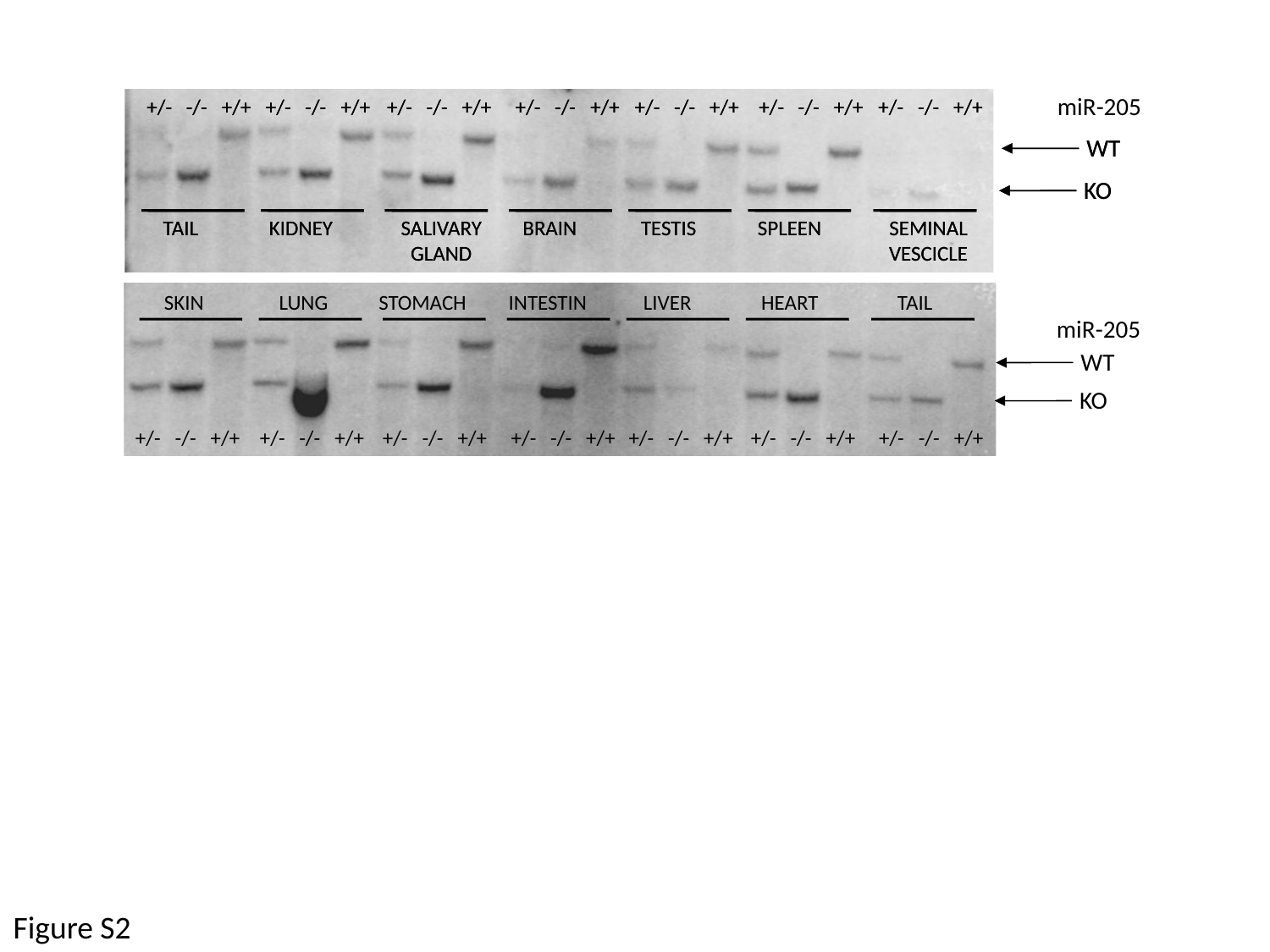

miR-205
+/-
-/-
+/+
+/-
-/-
+/+
+/-
-/-
+/+
+/-
-/-
+/+
+/-
-/-
+/+
+/-
-/-
+/+
+/-
-/-
+/+
+/-
-/-
+/+
+/-
-/-
+/+
+/-
-/-
+/+
+/-
-/-
+/+
+/-
-/-
+/+
+/-
-/-
+/+
+/-
-/-
+/+
WT
WT
KO
KO
TAIL
TAIL
KIDNEY
KIDNEY
SALIVARY GLAND
SALIVARY GLAND
BRAIN
BRAIN
TESTIS
TESTIS
SPLEEN
SPLEEN
SEMINAL VESCICLE
SEMINAL VESCICLE
SKIN
LUNG
STOMACH
INTESTIN
LIVER
HEART
TAIL
WT
KO
+/-
-/-
+/+
+/-
-/-
+/+
+/-
-/-
+/+
+/-
-/-
+/+
+/-
-/-
+/+
+/-
-/-
+/+
+/-
-/-
+/+
miR-205
Figure S2

## Slide 3
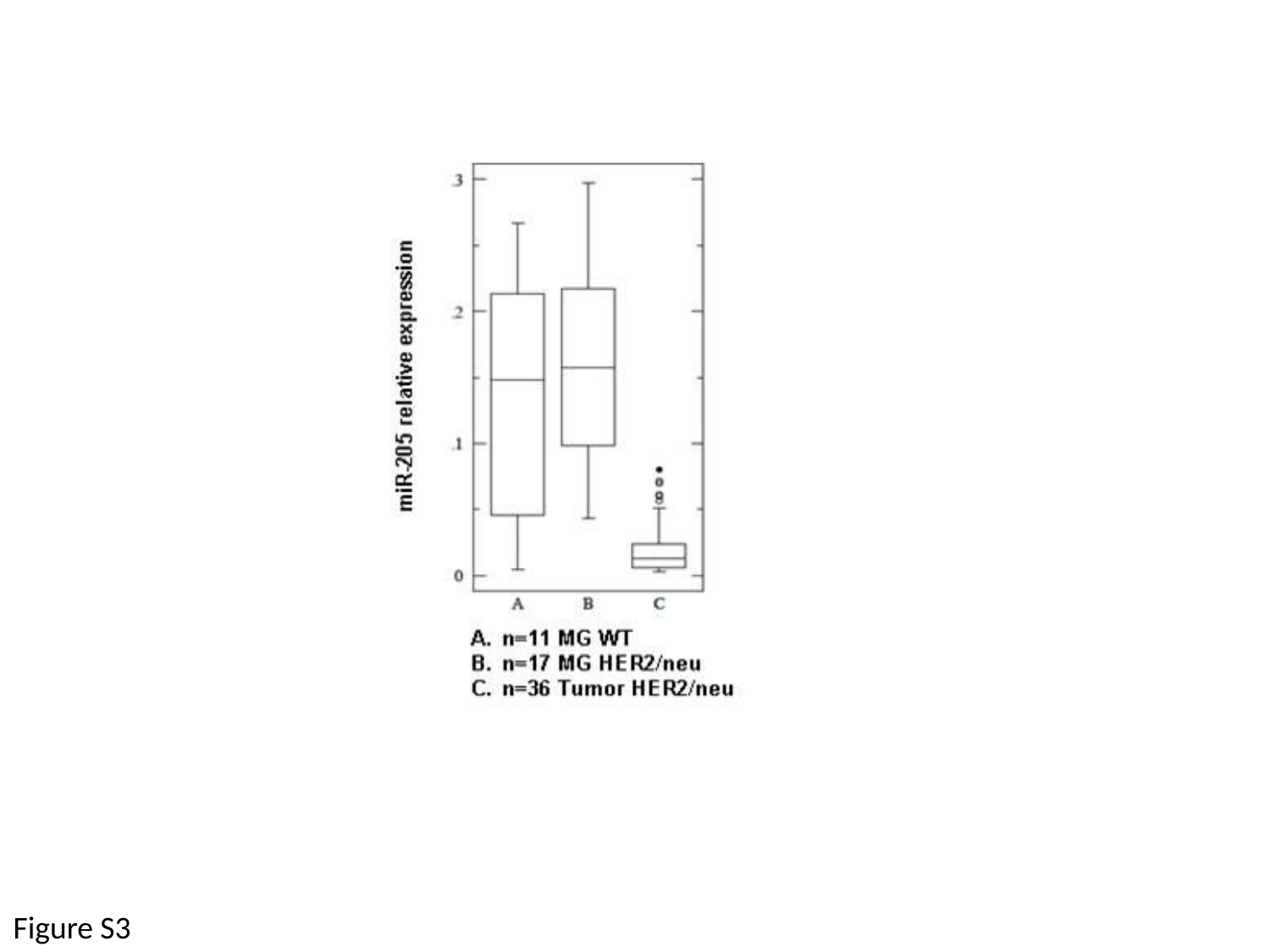

Figure S3
